# Oxidative-Stress-Mediated Epigenetic Dysregulation in Spermatogenesis: Implications for Male Infertility and Offspring Health

**DOI:** 10.3390/genes16010093

**Published:** 2025-01-17

**Authors:** Aris Kaltsas, Eleftheria Markou, Maria-Anna Kyrgiafini, Athanasios Zikopoulos, Evangelos N. Symeonidis, Fotios Dimitriadis, Athanasios Zachariou, Nikolaos Sofikitis, Michael Chrisofos

**Affiliations:** 1 Third Department of Urology, Attikon University Hospital, School of Medicine, National and Kapodistrian University of Athens, 12462 Athens, Greece; ares-kaltsas@hotmail.com; 2 Department of Microbiology, University Hospital of Ioannina, 45500 Ioannina, Greece; eleftheria.markou4@gmail.com; 3 Laboratory of Genetics, Comparative and Evolutionary Biology, Department of Biochemistry and Biotechnology, University of Thessaly, Viopolis, Mezourlo, 41500 Larissa, Greece; mkyrgiafini@uth.gr; 4 Obstetrics and Gynecology, Royal Devon and Exeter Hospital, Barrack Rd, Exeter EX2 5DW, UK; thanzik92@gmail.com; 5 Department of Urology II, European Interbalkan Medical Center, 55535 Thessaloniki, Greece; evansimeonidis@gmail.com; 6 Department of Urology, Faculty of Medicine, School of Health Sciences, Aristotle University of Thessaloniki, 54124 Thessaloniki, Greece; helabio@yahoo.gr; 7 Laboratory of Spermatology, Department of Urology, Faculty of Medicine, School of Health Sciences, University of Ioannina, 45110 Ioannina, Greece; azachariou@uoi.gr (A.Z.); nsofikit@uoi.gr (N.S.)

**Keywords:** oxidative stress, epigenetics, spermatogenesis, male infertility, reactive oxygen species, transgenerational inheritance

## Abstract

Male reproductive health is governed by an intricate interplay of genetic, epigenetic, and environmental factors. Epigenetic mechanisms—encompassing DNA methylation, histone modifications, and non-coding RNA activity—are crucial both for spermatogenesis and sperm maturation. However, oxidative stress, driven by excessive reactive oxygen species, disrupts these processes, leading to impaired sperm function and male infertility. This disruption extends to epigenetic modifications, resulting in abnormal gene expression and chromatin remodeling that compromise genomic integrity and fertilization potential. Importantly, oxidative-stress-induced epigenetic alterations can be inherited, affecting the health and fertility of offspring and future generations. This review investigates how oxidative stress influences epigenetic regulation in male reproduction by modifying DNA methylation, histone modifications, and non-coding RNAs, ultimately compromising spermatogenesis. Additionally, it discusses the transgenerational implications of these epigenetic disruptions and their potential role in hereditary infertility and disease predisposition. Understanding these mechanisms is vital for developing therapeutic strategies that mitigate oxidative damage and restore epigenetic homeostasis in the male germline. By integrating insights from molecular, clinical, and transgenerational research, this work emphasizes the need for targeted interventions to enhance male reproductive health and prevent adverse outcomes in progeny. Furthermore, elucidating the dose–response relationships between oxidative stress and epigenetic changes remains a critical research priority, informing personalized diagnostics and therapeutic interventions. In this context, future studies should adopt standardized markers of oxidative damage, robust clinical trials, and multi-omic approaches to capture the complexity of epigenetic regulation in spermatogenesis. Such rigorous investigations will ultimately reduce the risk of transgenerational disorders and optimize reproductive health outcomes.

## 1. Introduction

Male reproductive health is governed by a complex interplay of genetic, epigenetic, and environmental factors essential for spermatogenesis and sperm functionality. Epigenetic mechanisms, which involve heritable changes in gene activity without altering the DNA sequence, play a pivotal role in male fertility. Key processes such as histone modifications, DNA methylation, and the activity of non-coding RNAs ensure proper sperm maturation, chromatin organization, and the establishment of paternal genomic imprints necessary for successful fertilization and embryonic development [1].

Understanding oxidative stress in spermatogenesis is critical due to its profound implications for male fertility and reproductive health. Recent statistics indicate that male infertility affects approximately 7–10% of couples worldwide, with oxidative stress being a significant contributing factor [2]. This imbalance between reactive oxygen species (ROS) production and antioxidant defenses can impair sperm quality, including count, motility, morphology, and DNA integrity [3].

In the male reproductive system, ROS are produced through both endogenous and exogenous sources. Endogenous ROS primarily arise from metabolic processes such as mitochondrial activity within spermatozoa, particularly in the electron transport chain, and enzymatic activity involving NADPH oxidase and xanthine oxidase. In addition, infiltrating leukocytes during testicular inflammation or infection are significant contributors to ROS levels in the seminal plasma [4,5]. Exogenous sources include environmental toxins such as pesticides and heavy metals, as well as lifestyle factors like smoking, alcohol consumption, and poor diet. Exposure to heat stress and electromagnetic radiation has also been implicated in elevating ROS levels in testicular tissue [6,7,8].

Furthermore, studies have shown that oxidative stress is linked to various pathologies, such as diabetes and obesity, which exacerbate spermatogenic dysfunction [9,10]. Research has also demonstrated that oxidative stress induces apoptosis in testicular cells by disrupting the Bcl-2/Bax ratio, leading to decreased spermatogenesis and increased infertility rates [11,12].

Although these epigenetic processes are vital for normal sperm development, they are highly dynamic and vulnerable to external factors. Environmental stressors, including exposure to toxins, aging, and oxidative stress, can disrupt these regulatory mechanisms [13,14]. Oxidative stress causes direct cellular damage by oxidizing lipids, proteins, and nucleic acids, compromising DNA integrity, chromatin remodeling, and the activity of epigenetic enzymes such as DNA methyltransferases and histone-modifying proteins [15].

Recent research emphasizes the significant impact of oxidative stress on male fertility through its influence on epigenetic regulation. Beyond immediate detriments to sperm quality, oxidative-stress-induced epigenetic changes may have lasting consequences. Abnormal DNA methylation altered histone modification patterns, and dysregulated non-coding RNA expression have been linked to impaired sperm motility, defective chromatin structure, and increased DNA fragmentation, all of which contribute to infertility. Moreover, the activation of nuclear factor erythroid 2-related factor 2 (Nrf2) has been identified as a critical protective mechanism against oxidative stress in spermatogenesis [16,17]. The disruption of Nrf2 signaling exacerbates oxidative damage in the testes, highlighting the importance of this pathway in maintaining testicular health and function [10,16].

These changes may extend beyond the individual, with evidence suggesting that epigenetic alterations in sperm can be inherited by subsequent generations, potentially affecting gene expression and health in offspring [18]. For instance, the hypermethylation or hypomethylation of critical gene regions associated with oxidative stress have been linked to impaired spermatogenesis and a higher risk of genetic abnormalities in descendants [19,20].

Understanding the molecular pathways by which oxidative stress impacts epigenetic regulation in spermatogenesis is essential for developing interventions aimed at mitigating these effects. Mechanistic studies have revealed that ROS can directly modify epigenetic regulators, for instance, by oxidizing cysteine residues in DNA methyltransferases, leading to aberrant methylation patterns. Additionally, ROS interfere with histone acetyltransferases and deacetylases, altering chromatin accessibility and disrupting transcriptional programs essential for germ cell differentiation [21,22].

This manuscript aims to provide a comprehensive review of the impact of oxidative stress on epigenetic regulation in male fertility and its potential for transgenerational effects. By synthesizing insights from cellular, molecular, and clinical studies, it highlights how oxidative stress impairs male reproductive health and influences offspring outcomes. Additionally, this exploration emphasizes the need for therapeutic strategies to counteract oxidative stress and restore epigenetic balance in the male germline, thereby safeguarding paternal contributions to future generations.

## 2. Materials and Methods

### 2.1. Literature Search Strategy

A comprehensive literature search was conducted to gather peer-reviewed articles focused on the interplay between oxidative stress, epigenetic regulation, and male infertility. Three electronic databases—PubMed, Scopus, and Web of Science—were selected due to their extensive coverage of biomedical, life science, and multidisciplinary research. The searches covered publications from the inception of each database up to December 2024, ensuring that both foundational and recent investigations were included.

### 2.2. Search Terms and Eligibility Criteria

The following medical subject headings (MeSH) and keywords were used, either alone or in combination with Boolean operators (“AND”, “OR”): “oxidative stress”; “epigenetics”; “DNA methylation”; “histone modifications”; “non-coding RNAs”; “male infertility”; “spermatogenesis”; “sperm quality”; “transgenerational inheritance”. Articles were screened based on their relevance to the topic of oxidative-stress-induced epigenetic changes in male reproduction and potential transgenerational effects. To be included, studies needed to (i) present data or analyses directly relating to epigenetic modifications (e.g., DNA methylation, histone post-translational modifications, or non-coding RNA alterations) under conditions of oxidative stress, and (ii) focus on male germ cells, sperm parameters, or associated reproductive outcomes. Both original studies (clinical, experimental) and review articles were considered for completeness of the narrative. Publications not written in English, conference abstracts without sufficient methodological detail, and studies solely addressing female or somatic epigenetic changes were excluded.

### 2.3. Study Selection and Data Extraction

All search results were imported into a reference management software (EndNote 20) to facilitate the removal of duplicates. Titles and abstracts were independently screened by at least two authors (A.K. and E.M.). Any discrepancies regarding eligibility were resolved through discussion or by consulting a third author (M.C.). Key findings from each eligible study were subsequently synthesized under the main thematic categories of (i) epigenetic processes in spermatogenesis, (ii) oxidative-stress-related epigenetic disruptions, (iii) transgenerational effects, and (iv) clinical implications.

### 2.4. Quality Assessment

Although this review was designed primarily as a narrative synthesis, an informal assessment of methodological quality was performed by examining the clarity of each study’s objectives, the description of experimental or clinical methods, and the relevance of outcome measures. Studies with insufficient detail on experimental design or analytic methods were excluded, especially if they did not clearly report on oxidative stress markers or epigenetic parameters.

### 2.5. Data Synthesis

Relevant information from the included literature was grouped according to the type of epigenetic modification. Emerging themes were identified, such as how oxidative stress modifies DNA methylation patterns, histone marks, or non-coding RNA expression in spermatozoa. Conflicting results and gaps in knowledge were also highlighted to guide future directions. The final structure of the review was organized to reflect the interconnected topics of epigenetic regulation, environmental/lifestyle risk factors, transgenerational outcomes, and potential therapeutic approaches to counteract oxidative stress in male germ cells.

## 3. Epigenetic Mechanisms in Spermatogenesis and the Impact of Oxidative Stress

Spermatogenesis is a complex and meticulously regulated process that transforms primordial germ cells into mature spermatozoa capable of fertilization [23]. Epigenetic alterations are essential in coordinating this change by modifying gene expression without altering the underlying DNA sequence [24]. These alterations, including DNA methylation dynamics, histone modifications, and non-coding RNA regulation, are crucial for proper germ cell development, genomic imprinting, and the preservation of genomic integrity [25,26].

Furthermore, epigenetic regulation during spermatogenesis can be profoundly affected by oxidative stress, which arises from an imbalance between ROS production and the body’s antioxidant defenses. Elevated ROS levels can lead to lipid peroxidation, protein modifications, and DNA damage, all of which may disrupt normal gene expression and epigenetic patterns. Understanding the impact of oxidative stress on male fertility requires examining how it influences DNA methylation, histone modifications, and non-coding RNA expression [27].

Nonetheless, although numerous studies collectively emphasize the role of these epigenetic mechanisms in spermatogenesis, there remain gaps and contradictions in the literature, particularly concerning the degree to which human observations parallel animal models [26]. Some investigations in mice, for example, demonstrate pronounced epigenetic alterations resulting from oxidative stress; yet, corresponding human studies have reported more variable outcomes, likely due to confounding factors such as lifestyle, comorbidities, and age. Greater critical analysis of these divergent findings is, therefore, required to contextualize the epigenetic consequences of oxidative stress across different populations [28].

**Figure 1:**
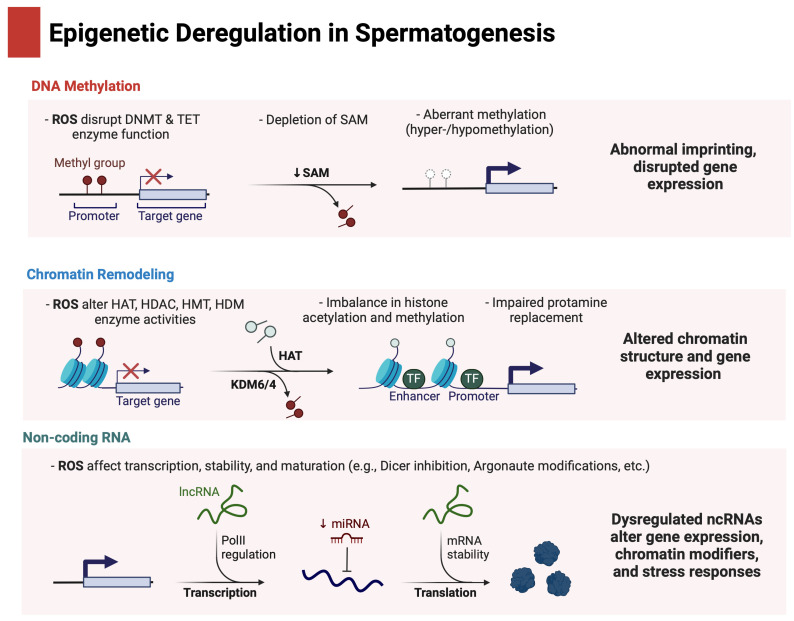
Epigenetic deregulation in spermatogenesis under oxidative stress. Created in BioRender. Kaltsas, A. (2025) https://BioRender.com/c15h647 (accessed on 28 December 2024).

Figure 1 below provides a concise overview of these epigenetic mechanisms, DNA methylation, chromatin remodeling, and non-coding RNA regulation, and illustrates how each can be disrupted by oxidative stress, potentially leading to impaired spermatogenesis and male infertility.

### 3.1. DNA Methylation Dynamics and Oxidative Stress

DNA methylation, the addition of a methyl group to the 5′ position of cytosine residues (often at CpG dinucleotides), plays a crucial role in regulating gene expression by altering chromatin structure and influencing transcription factor binding [29]. DNA methylation predominantly occurs within CpG islands, which are cytosine- and guanine-rich regions situated upstream of approximately 40% of mammalian genes [30,31]. The methylation status of CpG sites can significantly influence gene expression by blocking transcription factor binding or promoting the recruitment of transcriptional repressors, thus inhibiting transcription. Proper DNA methylation is crucial for several physiological processes, including X chromosome inactivation and the maintenance of chromatin stability [32].

During spermatogenesis, DNA methylation undergoes dynamic changes essential for germ cell differentiation and establishing paternal genomic imprints [33,34]. In early germ cell development, primordial germ cells (PGCs) experience global demethylation, effectively resetting the epigenetic state and erasing previous genomic imprints. As PGCs differentiate into spermatogonia, DNA methylation patterns are re-established, forming paternal-specific methylation profiles critical for genomic imprinting and gene regulation during spermatogenesis [35,36].

DNA methyltransferases (DNMTs) are responsible for establishing and maintaining these methylation patterns. DNMT1 maintains existing methylation marks during DNA replication, ensuring the faithful transmission of epigenetic information. DNMT3A and DNMT3B facilitate de novo methylation by adding methyl groups to unmethylated cytosines during development and cell differentiation. DNMT3L, lacking catalytic activity, acts as a co-factor to enhance DNMT3A and DNMT3B activity during germ cell development [37,38].

Studies have linked abnormal DNA methylation patterns in sperm to reduced semen quality and infertility [39,40]. Altered methylation correlates with impaired sperm motility, chromatin integrity, and DNA fragmentation. For instance, the hypermethylation of gene promoters such as *MTHFR*, *IGF2*, *H19*, *PLAG1*, and *SNRPN* has been associated with diminished sperm quality and an increased infertility risk, underscoring the importance of proper DNA methylation in male reproductive health [41,42].

Oxidative stress significantly impacts DNA methylation dynamics by affecting DNMT activity and expression. ROS can induce DNA damage, disrupting methylation processes by inhibiting DNMTs or recruiting DNA repair enzymes that modify methylation marks. For example, DNMT1 contains cysteine residues susceptible to oxidation, which can impair its enzymatic activity and result in aberrant DNA methylation patterns [43]. Additionally, ROS reduce the availability of the cofactor S-adenosylmethionine (SAM), a crucial cofactor for DNMT activity [44,45,46].

ROS can inhibit enzymes like methionine adenosyltransferase, which synthesizes SAM, and methionine synthase, which regenerates methionine. This diversion of methionine for cysteine production, needed to generate the antioxidant glutathione, further depletes SAM [47]. Prolonged ROS exposure, such as through hydrogen peroxide (H_2_O_2_), reduces SAM levels and increases glutathione, resulting in the hypomethylation of genomic elements like long interspersed nuclear element-1 (LINE-1) [48].

ROS also affect DNA demethylation by interfering with Ten-eleven translocation (TET) enzymes, which oxidize 5-methylcytosine to facilitate active demethylation [49]. Oxidative damage to TET enzymes or disruptions in their essential cofactors can hinder DNA demethylation, affecting gene expression [50,51]. Furthermore, oxidative-stress-induced inflammation can alter DNA methylation by modulating DNMT and TET enzyme activity [52,53].

Conversely, ROS can induce DNA hypermethylation by upregulating DNMT expression. Elevated ROS levels stimulate hypoxia-inducible factor 1-alpha (HIF-1α), which enhances DNMT1, DNMT3A, and DNMT3B expression, leading to global or site-specific DNA hypermethylation [54]. This upregulation leads to the global DNA hypermethylation or specific hypermethylation of CpG islands in genes such as SOD2, reducing their expression and contributing to cellular damage [55]. Furthermore, ROS can recruit DNMTs to DNA without altering their expression, with factors such as Snail promoting DNMT1 binding to gene promoters, resulting in hypermethylation and gene silencing [56].

These oxidative-stress-induced alterations in DNA methylation can result in abnormal gene expression, impacting critical physiological processes. Inadequacies in DNA methylation have been linked to various disorders, and such epigenetic changes can have significant implications for male reproductive health [57,58].

A number of discrepancies in the literature persist regarding the precise thresholds of oxidative stress at which DNA methylation patterns become significantly altered in humans as opposed to animal models. Some studies indicate that mild oxidative stress can already induce noticeable changes, while others report that only chronic or severe exposures are necessary to see consistent modifications. Addressing these inconsistencies requires standardized methodologies and larger population-based research to clarify the dose–response relationship between oxidative stress and DNA methylation in human sperm [58,59].

In summary, DNA methylation is critical in regulating gene expression during spermatogenesis. Oxidative stress disrupts these dynamics by damaging DNA and altering essential enzymes, leading to both hypo- and hypermethylation patterns. Although animal studies consistently demonstrate these disruptions, human data present more variability, underscoring the importance of refining experimental designs and controlling for confounding factors such as diet, age, and environmental exposures.

### 3.2. Histone Modifications, Chromatin Remodeling, and Oxidative Stress Effects

Histone modifications represent another critical epigenetic mechanism regulating gene expression during spermatogenesis. Post-translational modifications of histone proteins, such as acetylation and methylation, can alter chromatin structure, thereby influencing DNA accessibility and transcriptional activity. These modifications are essential for the proper progression of germ cells through the various stages of development [60,61].

Histone acetyltransferases (HATs) catalyze the addition of acetyl groups to lysine residues on histone tails, a process known as histone acetylation. This modification neutralizes the positive charge of histones, reducing their attraction to the negatively charged DNA and resulting in a more relaxed chromatin structure. This relaxation facilitates the binding of transcription factors and promotes gene expression. During the round spermatid stage, the hyperacetylation of histone H4 is particularly critical, as it is necessary for the transition from histones to transition proteins (TPs), which are essential intermediates in chromatin remodeling during sperm development [62,63].

Conversely, histone methylation, regulated by histone methyltransferases (HMTs), involves the addition of methyl groups to lysine or arginine residues. Depending on the residue and degree of methylation, this can either activate or repress transcription. For instance, methylation at H3K4 promotes transcription, while methylation at H3K9 and H3K27 is associated with gene silencing and heterochromatin formation. In spermatocytes, the precise control of H3K4 and H3K9 methylation is critical for regulating genes involved in meiosis and germ cell development [64,65].

Oxidative stress can profoundly disrupt histone modification patterns by altering the activity of histone-modifying enzymes. ROS can impair the balance between HATs and histone deacetylases (HDACs), leading to aberrant chromatin structures. For example, ROS can inhibit HATs or enhance HDAC activity, resulting in histone hypoacetylation, which tightens chromatin and suppresses gene expression [66]. ROS can also modify the core histones H3 and H4, especially on their exposed tails beyond the nucleosome. ROS influence both histone acetylation and methylation, which are among the most recognized modifications affected by oxidative stress [67,68].

Regarding methylation, ROS can interfere with both HMTs and histone demethylases (HDMs). These modifications can interfere with the proper progression of spermatogenesis by affecting the expression of genes crucial for germ cell development and maturation [69]. HMTs, such as lysine-specific and arginine-specific methyltransferases, require SAM as a methyl donor. ROS may diminish SAM availability by interfering with methionine metabolism, thus affecting HMT activity and resulting in improper histone methylation [70]. Additionally, ROS can inhibit the function of HDMs, like the Jumonji C domain-containing demethylases, which require Fe(II) and α-ketoglutarate as cofactors. Oxidative stress can oxidize Fe(II) to Fe(III), rendering the enzyme inactive and disrupting the dynamic regulation of histone methylation [71].

In the case of acetylation, ROS can disrupt the balance between HATs and HDACs. This imbalance can result in changes to chromatin structure, affecting the accessibility of DNA to transcription factors and consequently altering gene expression [72]. Oxidative stress may inhibit HAT activity or enhance HDAC activity, leading to the hypoacetylation of histone tails. This hypoacetylation increases the positive charge on histones, strengthening their interaction with the negatively charged DNA and resulting in a more condensed chromatin structure that represses gene transcription [71]. Moreover, ROS can affect class III HDACs, known as sirtuins (SIRT), which link lysine deacetylation with NAD⁺ hydrolysis. Alterations in NAD⁺ levels due to oxidative stress can impair sirtuin activity, further disrupting histone acetylation patterns [73].

Altered histone modification patterns can lead to chromatin compaction or relaxation at inappropriate genomic regions, resulting in disrupted gene regulation [74]. The consequences of oxidative-stress-induced histone modification changes include impaired chromatin remodeling, abnormal sperm development, and potential infertility. Understanding these effects is critical for developing interventions to mitigate the impact of oxidative stress on male reproductive health [75].

A hallmark of spermatogenesis is the dramatic chromatin remodeling that occurs during the transition from spermatids to mature spermatozoa. This process involves the replacement of histones with protamines, which facilitates the condensation of sperm chromatin into a highly compact structure. Initially, histones are replaced by transition proteins, which aid in the removal of histones and prepare the chromatin for protamination. The subsequent replacement of transition proteins with protamines results in a sixfold increase in DNA packaging efficiency compared to somatic cells. This extreme condensation is essential for protecting the paternal genome during transit and ensuring the integrity of genetic information passed to the offspring [76,77].

However, the failure of protamine replacement disrupts this critical compaction, leading to compromised chromatin integrity and a higher susceptibility to oxidative damage. Clinically, such inadequate protamination can result in elevated DNA fragmentation, reduced fertilization potential, and an increased risk of developmental issues in offspring [78,79]. By understanding the epigenetic and oxidative mechanisms that underlie protamine replacement failures, targeted interventions can be developed to preserve sperm quality and minimize the long-term reproductive consequences.

Failures in this process, such as insufficient histone acetylation or improper protamine replacement, can result in defective sperm maturation and compromised fertility. Understanding how oxidative stress affects histone modifications and chromatin remodeling is vital for developing therapies aimed at preserving male fertility and mitigating the long-term consequences of epigenetic dysregulation [80].

In summary, histone modifications and chromatin remodeling are essential for orchestrating gene expression in spermatogenesis. ROS disrupt these epigenetic regulators by altering enzyme activity and substrate availability, leading to potentially irreversible damage to sperm chromatin. Notably, some clinical and experimental studies diverge on whether acute or chronic oxidative stress has the more pronounced impact, highlighting the need for further inquiry into the precise timing, intensity, and duration of ROS exposure that leads to significant epigenetic disruption in human spermatogenesis.

### 3.3. Non-Coding RNAs in Sperm Development Affected by Oxidative Stress

Non-coding RNAs (ncRNAs), which do not encode proteins, play a crucial role in regulating gene expression at both the transcriptional and post-transcriptional levels. These include long non-coding RNAs (lncRNAs), microRNAs (miRNAs), and Piwi-interacting RNAs (piRNAs), all of which are essential for the differentiation, maturation, and function of germ cells during spermatogenesis [81,82].

MiRNAs are small RNA molecules, approximately 22 nucleotides long, that regulate gene expression by binding to complementary sequences in the 3′ untranslated region (UTR) of target mRNAs, leading to translational repression or mRNA degradation. The synthesis of miRNAs begins with the transcription of primary miRNA transcripts (pri-miRNAs) by RNA polymerase II [83,84]. Pri-miRNAs can originate from introns or untranslated regions of protein-coding genes, as well as from independent genes located in non-coding regions. During transcription, pri-miRNAs are produced with a 3′ polyadenylated tail and a 5′ 7-methylguanylate cap [85,86].

After transcription, pri-miRNAs undergo processing in the nucleus by the Drosha-DGCR8 complex, which cleaves them to generate precursor miRNAs (pre-miRNAs). The pre-miRNAs are then exported to the cytoplasm, where the Dicer-TRBP complex further processes them into mature miRNA duplexes. One strand of the duplex, the mature miRNA, is selected and incorporated into the miRNA-induced silencing complex (miRISC), which contains Argonaute (AGO) proteins. The miRISC binds to target mRNAs by recognizing sequences complementary to the miRNA seed region, leading to either translational repression or mRNA degradation [87].

MiRNAs play crucial roles in regulating spermatogenesis, sperm function, and other biological processes. They modulate the expression of genes essential for germ cell development and sperm function. The dysregulation of miRNAs has been associated with male infertility. Altered expression patterns of miRNAs have been observed in the spermatozoa of men with non-obstructive azoospermia (NOA) [88]. For instance, studies have identified the significant downregulation of miRNAs such as hsa-miR-34b-3p and hsa-miR-449a in NOA patients compared to fertile men. These miRNAs are predominantly expressed in the testis and have been implicated in apoptosis, cell proliferation, and differentiation, suggesting their crucial role in spermatogenesis [88,89].

Oxidative stress significantly modifies ncRNA expression and function, creating downstream effects that exacerbate cellular dysfunction. It can lead to the upregulation or downregulation of specific miRNAs and lncRNAs, thereby altering the expression of target genes involved in antioxidant defenses, inflammation, and apoptosis [90,91,92]. ROS can directly affect miRNA production by altering the expression and activity of enzymes such as Drosha and Dicer, and can also indirectly modulate miRNA expression by affecting ROS-sensitive transcription factors like NFκB, p53, Nrf2, and HIF1α [93,94,95]. Moreover, ROS can impact miRNA maturation by inhibiting key enzymes like Dicer and altering components of the miRISC complex, such as Argonaute proteins [96]. ROS may also stabilize typically transient miRNA strands, shifting the balance of stress-responsive pathways [97,98]. In addition, these alterations can facilitate transcription of stress-responsive lncRNAs, further influencing gene expression and potentially contributing to impaired spermatogenesis [99].

Altered miRNA expression profiles have been associated with male infertility. For instance, exposure to environmental toxins like dichlorodiphenyltrichloroethane (DDT) has been shown to induce changes in DNA methylation and non-coding RNA levels in sperm, potentially affecting subsequent generations through inheritance [100]. Studies have also identified the differential expression of miRNAs in Sertoli cells and spermatozoa, linking specific miRNAs to processes such as meiosis regulation, cell cycle progression, and sperm differentiation. Dysregulated miRNAs can influence gene expression patterns in sperm cells, potentially affecting the development and health of offspring [101].

Another class of small ncRNAs, known as piRNAs, typically range from 24 to 31 nucleotides in length and interact with Piwi proteins, a subclass of the Argonaute family. PiRNAs play a crucial role in silencing transposable elements in germ cells, thereby protecting genomic integrity by preventing insertional mutations. Unlike miRNAs, piRNA biogenesis does not involve the Drosha or Dicer enzymes, but is derived from single-stranded precursor RNAs through a “ping-pong” amplification cycle. In spermatogenesis, piRNAs are essential for germ cell development, regulating gene expression during meiosis and spermiogenesis [102,103].

Recent studies suggest that oxidative stress may affect piRNA expression and function, although the mechanisms are not fully understood. ROS can potentially alter piRNA biogenesis by affecting the proteins involved in the piRNA pathway, such as Piwi proteins and other associated factors. Oxidative stress may lead to the dysregulation of piRNAs, compromising their ability to silence transposable elements and maintain genomic integrity in germ cells [104]. The disruption of piRNA pathways due to oxidative stress could result in increased transposon activity, DNA damage, and mutations, ultimately affecting spermatogenesis and male fertility.

LncRNAs, which are transcripts longer than 200 nucleotides, regulate gene expression through various mechanisms, including chromatin remodeling, transcriptional interference, and acting as molecular scaffolds or decoys. In sperm development, lncRNAs are involved in the regulation of genes necessary for germ cell maturation and function. They contribute to chromatin organization and modulate the activity of transcription factors critical for spermatogenesis [105,106]. ROS can alter lncRNA transcription and stability, potentially promoting the expression of stress-responsive lncRNAs that interfere with normal germ cell development [91]. For instance, oxidative stress has been shown to increase the expression of lncRNAs like gadd7, which exacerbates ROS-induced stress responses and can lead to cell death [107]. In the context of spermatogenesis, changes in lncRNA expression due to oxidative stress could lead to aberrant gene expression patterns, affecting germ cell survival and differentiation [108]. Furthermore, specific lncRNAs have been identified that respond to oxidative stress by modulating apoptosis and necrosis pathways. For example, the lncRNA necrosis-related factor (NRF) facilitates oxidative-stress-induced necrosis by interacting with miR-873 and downregulating its target genes involved in cell survival [107]. Although these studies are primarily in somatic cells, similar mechanisms may operate in germ cells, where oxidative-stress-induced lncRNAs could influence spermatogenic cell fate decisions. Additionally, oxidative stress may affect lncRNAs involved in epigenetic regulation. ROS can influence the expression of lncRNAs that modulate chromatin-modifying enzymes, thereby indirectly affecting histone modifications and DNA methylation patterns crucial for spermatogenesis [109].

Oxidative stress can significantly impact the expression and function of non-coding RNAs—miRNAs, piRNAs, and lncRNAs—by modulating enzyme activities, transcription factors, and epigenetic landscapes. These alterations disrupt the finely tuned gene expression programs required for successful spermatogenesis, potentially leading to male infertility. Further research is needed to elucidate the specific mechanisms by which ROS influence piRNA and lncRNA pathways in germ cells, as understanding these processes is crucial for developing therapeutic strategies to mitigate oxidative-stress-induced ncRNA dysregulation and improving male reproductive health.

In summary, non-coding RNAs are integral to germ cell development, but their regulatory complexity poses challenges for clinical interpretation. While in vivo and in vitro models robustly demonstrate the ROS-induced dysregulation of miRNAs, piRNAs, and lncRNAs, some human cohort studies show less uniform results, highlighting a possible influence of genetic background, environmental variability, and population size. Future work should prioritize identifying key ncRNAs that consistently respond to oxidative stress and translating these findings to human fertility assessments.

## 4. Environmental and Lifestyle Factors Affecting Male Fertility

Male fertility is influenced by a combination of genetic, environmental, and lifestyle factors. Environmental exposures, lifestyle choices, and aging can increase oxidative stress and lead to epigenetic alterations that affect spermatogenesis and sperm quality. Understanding these factors is crucial for developing strategies to mitigate their impact on male reproductive health [75].

**Figure 2:**
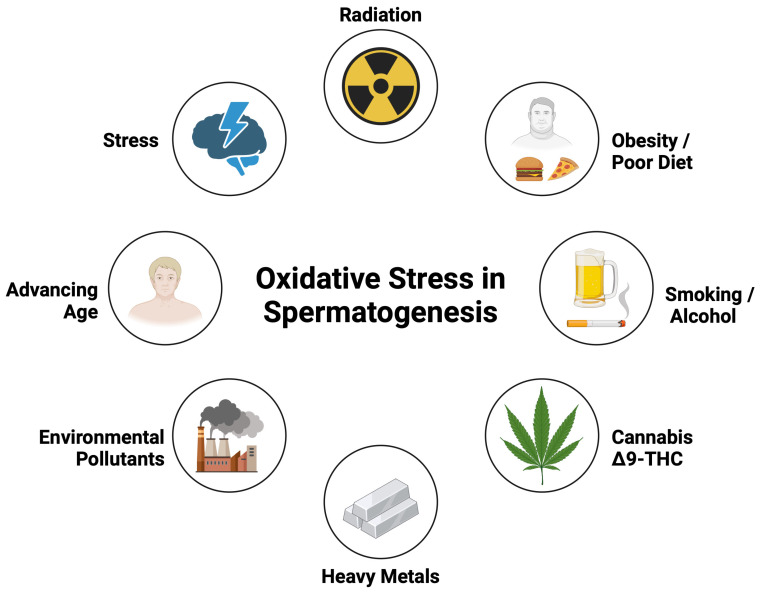
Environmental and lifestyle factors driving oxidative stress in spermatogenesis. Created in BioRender. Kaltsas, A. (2025) https://BioRender.com/n21f657 (accessed on 28 December 2024).

Figure 2 below provides an overview of key environmental and lifestyle factors, such as aging, pollutants, heavy metals, lifestyle habits, and radiation, that contribute to oxidative stress in spermatogenesis. By illustrating these influences together, it highlights the multifaceted ways in which external pressures can compromise sperm development through epigenetic dysregulation.

### 4.1. Age-Related Changes

Advancing age is associated with increased oxidative stress and the accumulation of DNA damage in sperm cells due to declining DNA repair efficiency. This results in reduced semen quality, decreased sperm motility, and elevated DNA fragmentation. Additionally, aging disrupts epigenetic marks, including DNA methylation and histone modifications, affecting gene expression crucial for spermatogenesis [110].

Age-related epigenetic alterations increase the risk of genetic disorders in offspring. Studies have shown that advanced paternal age correlates with poor semen quality, elevated DNA damage, and a higher likelihood of genetic diseases in children. These effects are largely driven by oxidative damage accumulation and weakened DNA repair systems in aging sperm [111,112].

Nevertheless, there remains debate regarding whether the same age thresholds apply universally across diverse populations, as certain studies report that men beyond 40 or 45 years show significant declines in sperm quality, while others posit a gradual decline beginning even earlier [113]. These inconsistencies underscore a need for longitudinal data that capture genetic background, lifestyle, and exposure histories to clarify the true effect of age on sperm epigenetic integrity [114,115].

### 4.2. Environmental Exposures

Exposure to environmental pollutants, heavy metals, and endocrine-disrupting chemicals (EDCs) has been associated with adverse effects on male fertility. Substances like bisphenol A (BPA), pesticides, and industrial compounds can induce oxidative stress, leading to increased ROS production and subsequent damage to sperm cells [116].

BPA, a widely used plasticizer, is known to promote oxidative stress and elevate ROS levels in the body. Exposure to BPA can significantly affect spermatogenesis, steroidogenesis, and induce apoptosis in germ and Sertoli cells. It disrupts the early stages of sperm development, compromises the blood–testis barrier, and alters the expression patterns of non-coding RNAs, ultimately impacting sperm quality. The reproductive outcomes of male exposure to BPA vary based on factors such as the route, dosage, duration of exposure, and developmental stage [117].

Exposure to Δ9-tetrahydrocannabinol (Δ9-THC), the primary psychoactive component of cannabis, can adversely affect the spermatogenesis by disrupting the endocannabinoid system. Δ9-THC has been shown to alter DNA methylation at specific gene loci in spermatozoa, potentially passing epigenetic modifications to the embryo upon fertilization. This highlights the potential risk of epigenetic disruptions affecting the offspring’s health [118].

Heavy metals such as lead, cadmium, mercury, and arsenic can disrupt hormonal regulation, induce oxidative stress, and cause testicular toxicity. These metals interfere with the hypothalamic–pituitary–gonadal axis, leading to endocrine imbalances that adversely affect sperm production and maturation. They can also cause DNA damage and epigenetic alterations in sperm cells, affecting gene expression patterns essential for spermatogenesis [119].

Although animal models show a strong causal link between these environmental agents and infertility, human studies often yield conflicting results. The inconsistencies may arise due to differing exposure levels, genetic predispositions, or interactions with multiple pollutants. Future studies employing larger sample sizes, refined exposure assessment methods, and the consideration of synergistic toxicities are crucial to resolve these discrepancies and support evidence-based policy decisions [120].

### 4.3. Lifestyle Influences

Lifestyle factors including diet, smoking, alcohol consumption, stress, obesity, and physical inactivity have significant impacts on male fertility. These factors often contribute to increased oxidative stress, leading to sperm dysfunction and altered epigenetic regulation [121].

Smoking and excessive alcohol intake generate ROS, causing oxidative damage to sperm cells. They are associated with decreased sperm count and motility, and increased DNA fragmentation [122]. Poor diet and obesity can exacerbate oxidative stress and disrupt hormonal balance, further impairing sperm quality [123].

Stress can influence hormonal levels and increase cortisol production, which may interfere with testosterone synthesis and spermatogenesis. Additionally, stress can elevate oxidative stress levels, contributing to sperm damage [124].

Physical inactivity and a sedentary lifestyle can lead to metabolic imbalances and increased oxidative stress, negatively affecting sperm parameters. Conversely, regular exercise has been shown to improve antioxidant capacity and enhance sperm quality [125].

In germ cells, lifestyle factors can induce epigenetic modifications, including alterations in histones and DNA methylation patterns. These changes may have transgenerational effects on offspring growth and health by influencing gene expression in sperm cells [126].

Quantitative data examining the relative contribution of these lifestyle factors remain somewhat limited, with some studies reporting that the cessation of smoking can restore sperm parameters by upwards of 10–20%, while others find negligible effects after controlling for age and other comorbidities. The variations highlight that more rigorous prospective studies are needed to determine precise dose–response relationships and the potential reversibility of epigenetic damage once harmful habits cease [127,128].

### 4.4. Environmental Radiation and Male Infertility

Male fertility and sperm production have been shown to suffer negative consequences from exposure to both ionizing and non-ionizing environmental radiation. Given the ubiquity of non-ionizing radiation in modern life, particularly from devices such as computers, smartphones, Wi-Fi routers, and microwave ovens, this is especially concerning [129].

Research suggests that the radiofrequency electromagnetic fields (RF-EMF) emitted by these devices can negatively impact sperm characteristics, including motility, morphology, and count. Exposure to RF-EMF may induce oxidative stress, leading to DNA damage, genomic instability, and alterations in several key cell signaling pathways, such as the mitogen-activated protein kinase (MAPK/ERK) and PI3K/Akt pathways, as well as the activation of NF-κB and disturbances in mitochondrial signaling. By interfering with these pathways, RF-EMF can modify gene expression, disrupt normal cell proliferation and apoptotic regulation, and, ultimately, compromise spermatogenesis and reduce sperm quality [130,131].

Moreover, prolonged exposure to RF-EMF has been associated with hormonal changes in the testes, potentially contributing to decreased testosterone levels and impaired reproductive function. RF-EMF exposure may lead to genotoxicity, genomic instability, and oxidative stress. The increasing use of RF technologies could be linked to adverse effects on sperm, induced by RF-EMF, and may be intricately associated with infertility [132].

While the exact mechanisms by which RF-EMF affects the male reproductive system remain unclear, evidence suggests the need for further investigation. Factors influencing the extent of abnormalities arising from RF-EMF exposure include the duration of exposure, proximity to the radiation source, intensity, density, and depth of penetration. Although antioxidants may offer superficial solutions to mitigate these effects, addressing the underlying issue of escalating electromagnetic pollution is essential for reducing its impact on male fertility [133].

Environmental and lifestyle factors play significant roles in influencing male fertility through mechanisms involving oxidative stress and epigenetic modifications. Addressing these factors through lifestyle changes, reducing exposure to environmental toxins, and implementing protective measures against radiation may help mitigate their adverse effects on reproductive health. Understanding the interplay between these factors and epigenetic regulation is crucial for developing therapeutic strategies aimed at improving male fertility and preventing the transgenerational transmission of epigenetic defects.

Research into human populations reveals a spectrum of results: while some studies point toward a direct correlation between mobile phone usage and reduced sperm quality, others find minimal or no effect after adjusting for confounding factors like heat exposure from devices or overall stress levels. This discrepancy signals a pressing need for standardized exposure metrics and improved epidemiological designs to consolidate current knowledge on RF-EMF hazards to fertility [134,135].

## 5. Transgenerational Effects and Offspring Health

The field of epigenetics has garnered significant scholarly attention due to the potential for epigenetic modifications to be inherited across generations, thereby influencing the health of descendants. Although most epigenetic changes are typically erased during germ cell development and fertilization, certain modifications, such as the methylation of imprinted genes, histone alterations, and the presence of non-coding RNAs, can be transmitted to offspring and affect gene expression [136]. Recent findings suggest that these mechanisms not only influence gene expression, but also regulate chromatin accessibility, further determining transcriptional outcomes in progeny. Additionally, variations in chromatin compaction have been identified as mediators of epigenetic inheritance, particularly in genes associated with stress responses and metabolic regulation [137].

Research indicates that alterations in the sperm epigenome and short non-coding RNAs in obese individuals can have a substantial impact on their children’s health [138]. For instance, a study using a mouse model found that the offspring of obese male mice exhibited hypomethylation in the imprinting control region (ICR) of the H19/IGF2 gene within their liver cells, mirroring the epigenetic state observed in the fathers’ sperm. These findings suggest that epigenetic changes in germ cells may contribute to paternal transmission of metabolic traits to offspring [139]. Moreover, the overexpression of the H19 transcript was found to enhance gluconeogenesis, implying that paternal obesity could alter gluconeogenesis and, consequently, the child’s metabolism through the dysregulated methylation of this gene [139]. Complementing these observations, altered levels of sperm-borne miRNAs, such as miR-29 and miR-34, have been implicated in modulating offspring metabolic pathways, suggesting another layer of epigenetic regulation influenced by paternal obesity [140].

Transgenerational inheritance mechanisms involve both DNA methylation and histone modifications. In mice, increased levels of tri-methylation at lysine 4 on histone H3 (H3K4me3) in sperm have been shown to regulate the expression of numerous genes associated with metabolic, inflammatory, and developmental processes in offspring [141]. Further studies reveal that histone variants, such as H3.3, retained at specific genomic loci in sperm, play an essential role in maintaining these modifications across generations, potentially safeguarding epigenetic marks during early embryonic development [142]. The potential to pass genetic alterations to progeny may be linked not only to obesity, but also to exposure to harmful agents. For example, the significant hypermethylation of sperm DNA and the differentially methylated region (DMR) of the delta-like homolog 1 (DLK1) gene has been detected in male smokers. In a murine model, similar modifications were observed, with male offspring exhibiting overexpression of the Dlk1 gene in their livers, leading to increased hepatic fat accumulation and altered responses to glucose tolerance tests. This suggests that epigenetic changes induced by cigarette smoking can be inherited by offspring, affecting their metabolic function [143]. In addition, exposure to pollutants such as endocrine-disrupting chemicals has been shown to induce similar heritable alterations, emphasizing the role of environmental toxicants in shaping epigenetic landscapes [144].

Dietary factors, as previously mentioned, are anticipated to significantly influence the future health of progeny. This process may impact not only the general health of children, but also their future fertility. Studies in mice have demonstrated that exposure to toxic chemicals can cause changes in testicular function that are transmitted to unexposed offspring. Researchers found that alterations in methylation patterns, non-coding RNAs, and messenger RNAs observed in the Sertoli cells of exposed mice were also present in the Sertoli cells of subsequent generations. This indicates that modifications in the epigenetic profile may contribute to fertility impairments and could explain the gradual decline in male fertility observed in recent decades [145]. Moreover, epigenetic reprogramming involving spermatogonial stem cells has been identified as a critical factor, with studies highlighting the persistence of altered chromatin states in the germline and the subsequent transmission of these changes to progeny [146].

In light of these transgenerational concerns, oxidative stress mitigation strategies for improving male fertility have evolved beyond simple recommendations for lifestyle modifications, with several clinical trials examining the efficacy of antioxidant supplementation. For instance, coenzyme Q10, vitamin E, selenium, and N-acetylcysteine have been tested in subfertile men to reduce ROS levels and improve sperm quality. Although some studies report modest improvements in sperm count or motility, heterogeneity in trial design, antioxidant dosage, and patient selection have resulted in inconsistent outcomes. Consequently, further large-scale well-controlled trials are needed to clarify optimal supplementation regimens and establish clinically meaningful endpoints [147,148].

Experimental epigenetic therapies—including histone deacetylase inhibitors and DNA methylation modulators—represent another frontier for countering oxidative-stress-induced epigenetic disruptions. Although these interventions have shown promise in certain cancer treatments, their direct application to restoring normal germline epigenetic profiles remains largely preclinical. More advanced epigenetic drugs might, in principle, reduce aberrant DNA methylation or histone modifications caused by ROS, thereby enhancing sperm function and potentially lowering transgenerational risks. However, the long-term safety of such therapies, especially if epigenetic modifications persist in subsequent generations, requires rigorous assessment [149,150,151].

Ethical implications also warrant consideration if transgenerational epigenetic interventions become viable. Should germline-targeted therapies extend beyond the individual and affect future progeny, robust ethical guidelines would be essential to balance the potential benefits of preventing inherited fertility issues with possible unforeseen outcomes in descendants. Addressing these ethical dimensions involves weighing consent, autonomy, and the broader societal impact of germline alterations, given that epigenetic changes could theoretically be passed to offspring who have not consented to such interventions [152].

Taken together, these findings underscore the complexity of transgenerational epigenetic inheritance and its far-reaching implications for reproductive health and disease risk. However, discrepancies among studies—particularly between well-controlled animal experiments and more heterogeneous human populations—call for larger longitudinal human cohorts to validate mechanistic insights. Additionally, the interplay between multiple environmental insults (e.g., combined exposure to heavy metals and endocrine disruptors) and paternal lifestyle factors (e.g., diet, exercise, stress) may induce epigenetic profiles that differ substantially across populations. Integrating quantitative data on these exposures and employing multi-omic approaches in future research will help resolve current inconsistencies and refine our understanding of how paternal environmental and lifestyle factors impact offspring health [153,154].

## 6. Limitations and Future Directions

There is growing interest in understanding how environmental and lifestyle factors influence epigenetic mechanisms and their subsequent effects on reproductive function and child health. Despite this growing interest, a critical gap exists in our knowledge of how epigenetic alterations impair human spermatogenesis, an area that has largely been overlooked in current research. Future studies must focus on the molecular pathways through which these epigenetic modifications disrupt spermatogenesis to address this gap [155].

The proposition that paternal exposure to oxidative stress can induce transgenerational epigenetic changes affecting male fertility in offspring is highly debated. The susceptibility of epigenetic reprogramming to environmental influences during critical periods of embryonic development challenges the robustness and broad applicability of this hypothesis. A primary limitation in the existing literature is the lack of definitive in vivo evidence establishing a direct causal link between paternal oxidative stress and heritable epigenetic modifications that influence male fertility. Although several studies have suggested such associations, the scarcity of robust in vivo data undermines the support for this hypothesis [156].

The phenomenon of transgenerational inheritance has been extensively studied, particularly in animal models, which provide more definitive conclusions about its potential for transfer across generations. However, human studies have yielded contradictory results. For example, the Pregnancy and Childhood Epigenetics (PACE) consortium found no association between paternal body mass index and changes in global methylation or imprinted DNA regions in children [157]. This inconsistency raises questions about the validity and general applicability of the hypothesis that paternal exposure to oxidative stress induces transgenerational epigenetic alterations affecting male fertility in offspring.

A notable limitation in current research is the lack of compelling in vivo evidence substantiating a causal link between paternal oxidative stress and transgenerational epigenetic modifications that impact male fertility. While several studies have suggested such a correlation, the absence of substantial in vivo data leaves the hypothesis inadequately supported. Accurately quantifying oxidative damage presents a significant challenge, as inconsistencies among commercial assays used to assess oxidative damage introduce potential artifacts and ambiguities in interpreting results. Due to limited and inconsistent data, the relationship between oxidative stress and male infertility remains unclear. Well-conducted clinical trials have not demonstrated significant therapeutic benefits of antioxidant treatments for male infertility, prompting questions about the direct causal relationship between oxidative stress and male fertility [158].

Furthermore, oxidative stress can damage the genetic and epigenetic information of germ cells, potentially impacting the health and well-being of future generations. Several factors contribute to oxidative stress in men, including inflammation in the male reproductive tract, the production of ROS by sperm mitochondria, and the dysregulation of enzymes involved in sperm capacitation. The balance between pro-oxidant and antioxidant factors during germ cell development plays a crucial role in determining how ROS generation affects oxidative stress. When evaluating the overall redox balance, the effectiveness of an individual’s antioxidant defenses is just as important as the excessive production of ROS [159].

Antioxidant supplementation has been explored as a treatment for infertility in both men and women. However, the effectiveness of these interventions has been limited by the lack of appropriate diagnostic criteria. Most studies have administered antioxidants to infertile patients without first assessing their oxidative stress levels. Consequently, there is an urgent need for straightforward diagnostic assays that can accurately measure oxidative stress levels, such as lipid peroxidation in blood and semen and oxidative DNA damage in spermatozoa [147].

Despite mounting evidence that oxidative stress may modulate epigenetic processes, significant methodological hurdles constrain current research. One key challenge is the limited reliability of many commercial tests for oxidative damage, which often introduce artifacts and ambiguities that hinder accurate interpretation. Furthermore, identifying specific epigenetic modifications that are genuinely causative rather than merely correlative requires robust in vivo models and careful control of confounding variables, including genetic predispositions and lifestyle factors. The dynamic nature of epigenetic (re)programming—particularly during critical developmental windows—further complicates longitudinal research designs. Standardized protocols for measuring oxidative stress markers such as lipid peroxidation, oxidative DNA damage, and antioxidant capacity are urgently needed to allow for meaningful cross-study comparisons and reproducibility. Without consensus on appropriate biomarkers and rigorous in vivo evidence linking paternal oxidative stress to heritable epigenetic changes, definitive conclusions about the role of oxidative stress in male fertility remain elusive [160].

Given these limitations, further research is essential to elucidate the complex interactions between oxidative stress, epigenetic modifications, and reproductive health. Specifically, future studies should aim to provide robust in vivo evidence and focus on the molecular pathways involved in epigenetic modifications that disrupt spermatogenesis. Additionally, the current literature reveals a paucity of human studies assessing the impact of epigenetic alterations caused by non-genetic male infertility on both the offspring of affected individuals and subsequent generations. Only through such investigations can we determine whether an epigenetic change inherited by children can persist into future generations, potentially evolving into a novel genetic trait [161].

While existing studies offer valuable insights, significant limitations hinder a comprehensive understanding of the relationship between oxidative stress, epigenetic modifications, and male fertility. Overcoming these challenges will enhance the comprehension of ROS formation and its effects on male reproductive health, ultimately contributing to improved interventions and therapeutic strategies.

## 7. Conclusions

Epigenetic regulation is fundamental to spermatogenesis, with DNA methylation dynamics, histone modifications, and non-coding RNAs intricately controlling the development of mature spermatozoa. Oxidative stress disrupts these epigenetic mechanisms by inducing DNA damage, altering histone modification patterns, and modulating non-coding RNA expression, leading to impaired sperm function and male infertility. Environmental and lifestyle factors, including aging, exposure to pollutants, unhealthy habits, and radiation, exacerbate oxidative stress, contributing to epigenetic alterations that not only affect individual fertility, but may also have transgenerational implications. While animal studies have demonstrated the potential for epigenetic modifications to impact offspring health and fertility, human studies are limited and often present contradictory findings.

Given these complexities, advancing the understanding of the molecular pathways through which oxidative stress influences epigenetic regulation in human spermatogenesis is imperative. Future research should focus on providing robust in vivo evidence, developing precise diagnostic tools to assess oxidative damage and epigenetic alterations, and exploring effective therapeutic interventions. Addressing these gaps will enhance the understanding of the interplay between oxidative stress, epigenetics, and male reproductive health, ultimately contributing to improved fertility outcomes and the prevention of adverse transgenerational effects.

## Data Availability

No new data were created or analyzed in this study. Data sharing is not applicable to this article.
